# A Major *Mycobacterium tuberculosis* outbreak caused by one specific genotype in a low-incidence country: Exploring gene profile virulence explanations

**DOI:** 10.1038/s41598-018-30363-3

**Published:** 2018-08-08

**Authors:** Dorte Bek Folkvardsen, Anders Norman, Åse Bengård Andersen, Erik Michael Rasmussen, Troels Lillebaek, Lars Jelsbak

**Affiliations:** 1 International Reference Laboratory of Mycobacteriology, Statens Serum Institut, Copenhagen, Denmark; 2 Department of Biotechnology and Biomedicine, Technical University of Denmark, Kgs. Lyngby, Denmark; 3 Department of Infectious Diseases, Copenhagen University Hospital, Rigshospitalet, Copenhagen, Denmark; 4 Research Unit for Infectious Diseases, Department of Clinical Research, University of Southern Denmark, Odense, Denmark

## Abstract

Denmark, a tuberculosis low burden country, still experiences significant active *Mycobacterium tuberculosis* (Mtb) transmission, especially with one specific genotype named Cluster 2/1112–15 (C2), the most prevalent lineage in Scandinavia. In addition to environmental factors, antibiotic resistance, and human genetics, there is increasing evidence that Mtb strain variation plays a role for the outcome of infection and disease. In this study, we explore the reasons for the success of the C2 genotype by analysing strain specific polymorphisms identified through whole genome sequencing of all C2 isolates identified in Denmark between 1992 and 2014 (n = 952), and the demographic distribution of C2. Of 234 non-synonymous (NS) monomorphic SNPs found in C2 in comparison with Mtb reference strain H37Rv, 23 were in genes previously reported to be involved in Mtb virulence. Of these 23 SNPs, three were specific for C2 including a NS mutation in a gene associated with hyper-virulence. We show that the genotype is readily transmitted to different ethnicities and is also found outside Denmark. Our data suggest that strain specific virulence factor variations are important for the success of the C2 genotype. These factors, likely in combination with poor TB control, seem to be the main drivers of C2 success.

## Introduction

There is increasing evidence that, in addition to environmental factors^1^, drug resistance^2^ and human genetics^3^, strain variation in members of the *Mycobacterium tuberculosis* Complex (MTBC) plays a key role in the outcome of tuberculosis (TB) infection and disease^4,5^. Hence, there is a need to better understand the global diversity of MTBC in order to determine whether and how this diversity has relevance for TB control. Molecular epidemiology studies have demonstrated a diverse *Mycobacterium tuberculosis* (Mtb) population structure but also the existence of specific dominant clonal lineages with epidemic behaviour. This implies that some strains may have acquired functional advantages (over others) in their ability to transmit and cause disease^6^. One explanation could be the increased transmission of Drug-Resistant TB^7^, which demonstrates the ability of the bacterium to adapt to antibiotic pressure. However, dominant lineages without resistance exist^8^, and the specific relationship between the genetic background of the different Mtb lineages and their clinical phenotypes remains far from understood^9^.

In Denmark, one specific clonal strain has increased dramatically. This outbreak strain, “Cluster 2/1112–15” (hereafter, just C2), was first identified in 1992 in 8 patients. Over the last 25 years, the strain has caused disease in more than 1000 individuals, and is now the predominant outbreak strain in Scandinavia. Additionally, in 2001, C2 was transmitted to Greenland, adding to an existing heavy TB burden in this region^10^.

We have previously characterized the C2 outbreak using whole genome sequencing (WGS) on a sparse time-series consisting of 115 isolates (five from each of the years 1992–2014) and shown that it was a clonal outbreak belonging to MTBC lineage 4.8, with 2 discernible phylogenetic clades, a major and a minor, and a most common recent ancestor dating back to 1959 (95% CI 1944–1973), pointing to its introduction into Denmark sometime after the Second World War^11^. The closest known related strains were found to originate in Russia, but were separated from C2 by at least 200 years. Therefore, although the exact journey of C2 into Denmark remains elusive, our initial analysis raises the question, whether the current success of the C2 outbreak is attributable to a unique genetic virulence profile acquired prior to its introduction to Denmark. In order to investigate the potential biological backgrounds for the success of this Mtb strain, we extend our WGS analysis to all available C2 isolates identified between 1992 and 2014 to pinpoint all universally preserved mutations to allow a detailed analysis of all C2-specific polymorphisms. As the definition of virulence is still widely discussed, we use the terms virulence and success interchangeably.

## Results

### C2 in Denmark

We extended our WGS to include all C2 Mtb isolates identified in the Kingdom of Denmark from 1992 to 2014 through either Mycobacterial Interspersed Repetitive Units-Variable Number of Tandem Repeats (MIRU-VNTR) or Restriction Fragment Length Polymorphism (RFLP), respectively. Initially, this comprised 989 isolates, but this figure was later reduced to 952 isolates from 892 patients, due to 8 strains having first been misidentified as Cluster-2 or 1112–15, and 28 strains being excluded due to lack of growth (*n* = 12) or insufficient sequence coverage (*n* = 18). The C2 strains were found in patients with different nationalities and ethnicities (630 Danish-born (DB), 217 Greenlandic-born (GB) and 45 foreign-born (FB) (from Africa, Middle East, Asia and Europe, own data)). All Mtb strains had been susceptibility tested for the four standard drugs identifying only one strain resistant to isoniazid and another strain resistant to pyrazinamide. The median coverage of the 952 strains was 39.9× [IQR: 28.5–57.6].

### Genomic analysis of C2

Analysing the set of 952 isolates, we identified 1309 high quality SNPs, out of which 414 (Supplementary Table S1) were determined to be present in all C2 strains against the H37Rv background, hereafter referred to as monomorphic SNPs. The remaining 895 mutations arose during the C2 outbreak out of which 81 SNPs (9%) occurred in 5 or more strains. Of the 414 identified monomorphic SNPs, 234 (57%) were non-synonymous (NS), 133 (32%) were synonymous, and 47 (11%) were intergenic, corresponding to an overall dN/dS ratio of 0.64.

Of the 234 NS monomorphic SNPs, 58 were found in genes involved in cell wall and cell processes, 52 in genes involved in intermediary metabolism and respiration, 51 in conserved hypotheticals, 32 in genes involved in lipid metabolism, 16 in genes involved in information pathways, 11 in genes involved in virulence, detoxification or adaptation, 11 in genes involved in regulatory proteins, 2 in genes involved in insertion sequences and phages and 1 in a gene with unknown function. We found SNPs in 1–13% of the total numbers of genes in the different categories according to Tuberculist (Table 1). It is important to note that genes encoding enzymes important for cell wall and cell processes and intermediary metabolism and respiration are present with a high number of genes in Mtb^12^, we did however, find more SNPs in this category than expected (Table 1).

**Table 1:** SNPs in C2 distributed in different functional groups according to TubercuList.

Functional group	C2 SNPs	TubercuList	% of genes we found SNPs	Expected^a^	Expected # of SNPs^b^
Information pathways	16	242	6,61	0,06	14,66
Cell wall and cell processes	58	772	7,51	0,20	46,76
Intermediary metabolism and respiration	52	936	5,56	0,24	56,70
Virulence, detoxification, adaptation	11	239	4,60	0,06	14,48
Conserved hypotheticals	51	1042	4,89	0,27	63,12
Regulatory proteins	11	198	5,56	0,05	11,99
Lipid metabolism	32	272	11,76	0,07	16,48
Insertion seqs and phages	2	147	1,36	0,04	8,90
Unknown	1	15	6,67	0,00	0,91

^a^Number of genes in that category divided by number of genes in total, all according to TubercuList.

^b^Expected times total number of SNPs in C2.

### Universally conserved single-nucleotide polymorphisms in C2

**Figure 1:**
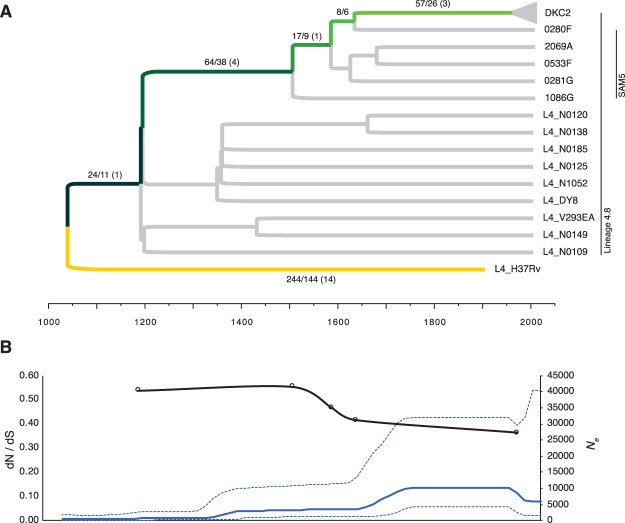
Distribution and overall selective pressure of monomorphic SNPs conserved in 952 isolates of the *M.tb* DKC2 outbreak. (**A**) Maximum clade credibility phylogeny inferred from 2414 SNPs on 10 representative genomes from a global MTBC collection^13^, five related genomes from Samara, Russia (SAM5)^34^ and four representative strains from the DKC2 outbreak using BEAST with a fixed molecular clock (5.0 × 10^−8^ SNPs/genome position/year) and a Bayesian Skyline population model. Green colored branches indicate the phylogenetic path that SNPs accumulated prior to the DKC2 outbreak have followed, while the yellow branch are monomorphic SNPs that have instead been accumulated in the H37Rv reference strain. Number of monomorphic SNPs per branch (Total SNPs/Non-synonymous SNPs) are displayed on respective branches. Numbers in parenthesis indicate distribution of non-synonymous SNPs in 23 genes previously associated with *M.tb* virulence. (**B**) Median effective population size (*N*_*e*_) derived from Bayesian skyline plot (blue line – 95% HPD range is indicated with stippled lines) and the calculated dN/dS ratio of monomorphic SNPs accumulated in the DKC2 clade from specific time points.

Out of the 414 SNPs (Supplementary Table S1), 244 (59%) were universally conserved among reference strains from a global collection of MTBC strains^13^, meaning that these differences likely stem from mutations arising in the reference strain H37Rv, since the two MTBC lineages 4.8 and 4.9 diverged. Furthermore, 24 SNPs were previously identified as universally conserved among strains belonging to MTBC lineage 4.8^14^ and 89 SNPs were also found to be conserved among the five strains from Samara, Russia (SAM5), previously identified as being more closely related to C2 than all other global strains from MTBC lineage 4.8 (Fig. 1A)^11^. Thus, 57 SNPs (14%) were uniquely conserved among C2 (26 synonymous, 26 non-synonymous and 5 intergenic). Accordingly, the dN/dS ratio decreased significantly, from 0.64 to 0.36, as more and more distantly related strains were removed from the analysis, resulting in much stronger purifying selection (Fig. 1B).

### Potential virulence factors

The NS monomorphic SNPs were used to investigate for potential virulence factors. This was done by searching the literature for association between any of the genes in which we found NS SNPs and virulence. Of the 234 NS monomorphic SNPs, 23 were found in genes previously described to be involved in Mtb virulence (Table 2). Out of the 57 SNPs conserved in C2, we found three non-synonymous mutations in genes previously associated with virulence.

**Table 2:** List of virulence associated monomorphic SNPs in C2.

Position	Variant	Locus	NT change	Lineage in which SNPs arose	AA change	Annotation	Functional category	Reference
55553	C- > T	ponA1 (Rv0050)	C1891T	H37Rv	P631S	Probable bifunctional penicillin-binding protein 1 A/1B PonA1 (murein polymerase) (PBP1): penicillin-insensitive transglycosylase (peptidoglycan TGASE) + penicillin-sensitive transpeptidase (DD-transpeptidase)	cell wall and cell processes	Kieser *PLOS Pathogens* 2015^50^
206339	T- > C	mce1F (Rv0174)	T1109C	L4.9	L370P	Mce-family protein Mce1F	virulence, detoxification, adaptation	Mikheecheva *GBE* 2017^35^
290633	T- > G	htdX (Rv0241c)	A23C	SAM5	K8Q	Probable 3-hydroxyacyl-thioester dehydratase HtdX	intermediary metabolism and respiration	Gurvitz *Journal of Bacteriology* 2009^51^
686972	T- > C	mce2A (Rv0589)	T152C	L4.9	F51S	Mce-family protein Mce2A	virulence, detoxification, adaptation	Mikheecheva *GBE* 2017^35^
755122	C- > T	mazE2 (Rv0660c)	G105A	C2	R35H	Possible antitoxin MazE2	virulence, detoxification, adaptation	Maisonneuve *PNAS* 2011^32^
775639	T- > C	mmpL5 (Rv0676c)	A2843G	H37Rv	I948V	Probable conserved transmembrane transport protein MmpL5	cell wall and cell processes	Wells *PLOS Pathogens* 2013^52^
852910	C- > T	phoR (Rv0758)	C515T	H37Rv	P172L	Possible two component system response sensor kinase membrane associated PhoR	regulatory proteins	Mikheecheva *GBE* 2017^35^
1037911	C- > T	pstA1 (Rv0930)	C913T	H37Rv	R305*	Probable phosphate-transport integral membrane ABC transporter PstA1	cell wall and cell processes	Mikheecheva *GBE* 2017^35^
1100234	T- > C	pepD (Rv0983)	T1169C	H37Rv	L390P	Probable serine protease PepD (serine proteinase) (MTB32B)	intermediary metabolism and respiration	Mikheecheva *GBE* 2017^35^
2211477	G- > C	mce3B (Rv1967)	G877C	SAM5	D293H	Mce-family protein Mce3B	virulence, detoxification, adaptation	Mikheecheva *GBE* 2017^35^
2216443	C- > A	mce3F (Rv1971)	C1187A	L4.9	A396E	Mce-family protein Mce3F	virulence, detoxification, adaptation	Mikheecheva *GBE* 2017^35^
2507254	G- > A	ptpA (Rv2234)	G109A	L4.8	A37T	Phosphotyrosine protein phosphatase PtpA (protein-tyrosine-phosphatase) (PTPase) (LMW phosphatase)	regulatory proteins	Mikheecheva *GBE* 2017^35^
2868793	C- > T	vapB19 (Rv2547)	C188T	SAM5	T63I	Possible antitoxin VapB19	virulence, detoxification, adaptation	Mikheecheva *GBE* 2017^35^
3137058	G- > A	vapB22 (Rv2830c)	C168T	L4.9	A56V	Possible antitoxin VapB22	virulence, detoxification, adaptation	Mikheecheva *GBE* 2017^35^
3292720	T- > C	pks1 (Rv2946c)	A3635G	SAM5	K1212E	Probable polyketide synthase Pks1	lipid metabolism	Mikheecheva *GBE* 2017^35^
3293677	G- > C	pks1 (Rv2946c)	C2678G	C2	L893V	Probable polyketide synthase Pks1	lipid metabolism	Mikheecheva *GBE* 2017^35^
3296843	A- > G	pks15 (Rv2947c)	T999C	H37Rv	V333A	Probable polyketide synthase Pks15	lipid metabolism	Gautam *Infectious Diseases* 2017^53^
3453123	G- > A	Rv3087	G199A	C2	G67S	Possible triacylglycerol synthase (diacylglycerol acyltransferase)	lipid metabolism	Mikheecheva *GBE* 2017^35^
3518555	A- > G	nuoG (Rv3151)	A1810G	L4.9	T604A	Probable NADH dehydrogenase I (chain G) NuoG (NADH-ubiquinone oxidoreductase chain G)	intermediary metabolism and respiration	Mikheecheva *GBE* 2017^35^
3826684	C- > T	vapC47 (Rv3408)	C137T	H37Rv	S46L	Possible toxin VapC47. Contains PIN domain.	virulence, detoxification, adaptation	Mikheecheva *GBE* 2017^35^
4055801	G- > A	espA (Rv3616c)	C576T	L4.9	T192I	ESX-1 secretion-associated protein A, EspA	cell wall and cell processes	Mikheecheva *GBE* 2017^35^
4210274	A- > G	tcrY (Rv3764c)	T737C	L4.9	C246R	Possible two component sensor kinase TcrY	regulatory proteins	Parish *Infection and Immunity* 2002^39^, Bhattacharya *Biochimie* 2010^38^
4288850	C- > T	mmpL8 (Rv3823c)	G2681A	SAM5	V894M	Conserved integral membrane transport protein MmpL8	cell wall and cell processes	Mikheecheva *GBE* 2017^35^

## Discussion

In this study, we analysed a major cluster of Mtb isolates (C2) associated with a TB outbreak with significant ongoing Mtb transmission for universally conserved genetic traits. The outbreak was initially confined to the capital city Copenhagen, predominantly in the inner city among socially marginalized persons, but subsequently transmitted all around the Danish kingdom, including Greenland, and to neighbouring countries. Clinical TB is influenced by variability in the host’s genetic background, immune status, diet, social, and environmental factors^15,16^ but little is known about the bacterial factors, especially genetic diversity in bacterial virulence factors that contribute to variable host responses.

The lifetime risk of developing active TB, when infected latently with Mtb is around 12%^17^. The reasons why some are more prone to develop TB, has been discussed widely, and a number of risk factors, such as HIV infection and immunosuppression, social factors, incarceration, or being a drug abuser, have been described^18^. Human genetic variation has also been suggested to play a role in success of TB^3,19^. The success of C2, however, does not seem to be strongly influenced by human genetic factors, as it is found in Denmark among different nationalities and ethnicities as diverse as ethnic Danes and ethnic Greenlanders.

**Figure 2:**
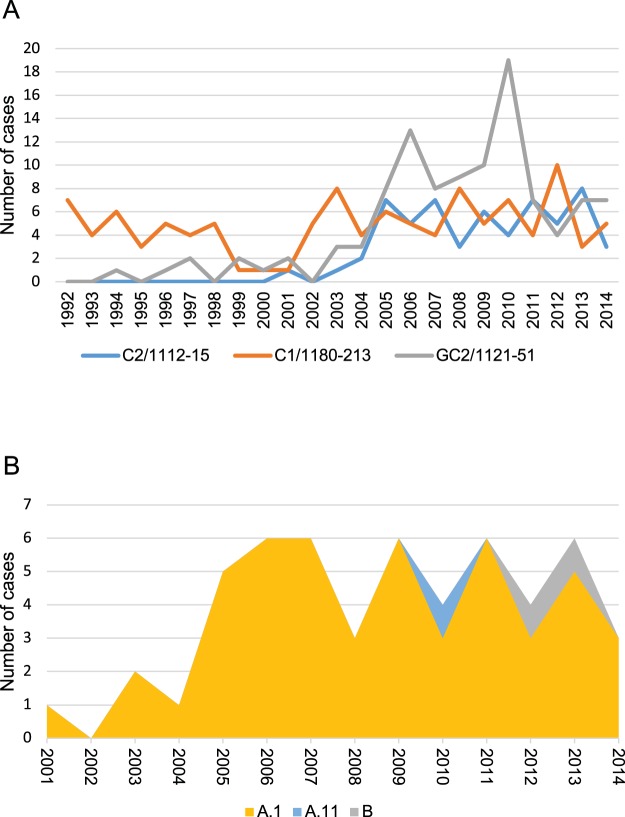
Distribution of the most frequent genotypes and of the C2 subgroups in Greenland. (**A**) Comparison of the number of cases among the three most frequent TB genotypes reported in Greenland 1992–2014. (**B**) Abundance of the different epidemic subgroups within the C2 genotype found in Greenland 2001–2014. Subgroup designations are from a previous article^11^.

It is likely that the main contributor to the success of the C2 strain is a lack of TB control and social problems, as is reported from other settings^8^. However, in Greenland, a total of 80 different MIRU-VNTR genotypes have been observed between 1992 and 2014. Of these, only 30 clusters with at least one other strain, and only 13 of these clusters have been seen in more than 10 patients, 3 of which are more abundant than C2. The most frequent of these, was found primarily in a remote setting in East Greenland^20^, and was therefore excluded from this comparison. In 2001, C2 was introduced and is spreading successfully in Greenland, a country already fighting an existing heavy TB-burden, suggesting that this particular strain, as well as the GC2 subtype^21^, may have some advantage over the many subtypes introduced in the same period (Fig. 2A). The majority of the C2 cases from Greenland belonged to the major-lineage subgroup A.1 (50/53, Fig. 2B) and between 2004 and 2014, there has been 2–8 cases a year in this small country, approximately the same number of cases as a historically big cluster in Greenland, the C1^22,23^ (Fig. 2A). As the vast majority of these cases belong to the same subgroup (Fig. 2B) it is a clear indication that C2 in Greenland stems from a single rather than several independent introductions of the strain.

The previously reported C2 mutation-rate of 0.24 SNPs/genome/year^11^ correlates well with previous findings^24–26^ and is in fact lower than some other findings^27,28^, indicating that the success of C2 is unlikely to have been caused by hypermutation. It has previously been reported that lineage 4 has a lower mutation-rate than lineage 2^5^, which in several studies reported as the most frequent^20,25,27,29^. Furthermore, as lineage 4 is not as prone to resistance as the Beijing lineage, some other factors must contribute to its worldwide success.

The overall dN/dS ratio of 0.64 also correlates well with previous findings^8,30^ and does not seem to suggest positive selection prior to the introduction of C2 into Denmark. In fact, there was a clear trend towards strong purifying selection (decrease in dN/dS ratio) over the last thousand years (Fig. 1B). This is most likely, as suggested by Pepperell *et al*., attributable to explosive growth in the human population size over this period^31^.

SNPs were found in all categories of functional genes. Most were found in genes involved in cell wall and cell processes and intermediary metabolism and respiration, which is in accordance with these categories being the most frequent^12^, however, we did find more SNPs than expected. High-throughput sequencing has yielded functional genomic data for many organisms, but a large proportion of the genes are labelled “hypotheticals” or “unknown”. For Mtb, it is the case for 27% percent of the genes (1057/3863)^12^ and it is speculated if better understanding of these genes might lead to a better understanding of virulence. We found 53 monomorphic NS SNPs in these undescribed genes. Even though it is less than the 68 expected (Table 1), until a better understanding of these genes is obtained, we can only speculate if they have a role in the success.

When searching the literature for virulence linked to certain genes, we could find reports of virulence for 23 of the genes with NS monomorphic SNPs seen in C2. Three of these SNPs, were found exclusively in C2 (Table 2). Examples include the *mazE2* gene, a toxin-antitoxin (TA) gene, reported to help with inducing dormancy and persistence of the bacteria^32^. Another example is the *pks1/15* gene, where an intact gene is reported to contribute to virulence by suppressing the human innate immune response^33^. Another 4 of the 23 SNPs reported to be involved in virulence, were found only in C2 and in the closest relative, the SAM5 group^11,34^ (Table 2). Among these are the MCE family proteins that play a role in adhesion and invasion of and survival inside macrophages^35^. Furthermore, when cloned into a non-pathogenic strain of *E. coli*, they gave *E. coli* the ability to enter and survive in mammalian cells, including macrophages^36,37^.

We also observed a monomorphic SNP in the *tcrY* gene. Interestingly, an additional mutation in this gene is found among the 81 most abundant SNPs observed within the C2 outbreak. In fact, this mutation is present in 844 out of 864 (98%) of all C2 major lineage strains, and could therefore be a contributing factor to the much higher number of strains in the major- than in the minor lineage (85 strains). Mtb holds 12 two component regulatory systems that enables the bacteria to respond to different external stress indicators, the *tcrXY* system is one of these and has been suggested to be involved in regulating the genes required for suppressing intracellular growth^38^. Knockout of this gene has resulted in increased virulence in and shorter survival time for SCID mice^39^.

The present study holds a number of limitations. As previously mentioned, a consensus of the definition of virulence has not been obtained and we here use the term interchangeably with “success”. In our literature search we looked for studies that link certain genes with virulence, not genes that are in the functional category “virulence” (Table 2). One approach to experimentally test for virulence, is to measure growth, either *in vitro* or *in vivo*^40^. This is outside the scope of this study. Furthermore, it has been suggested that repetitive regions, such as *pe-, ppe-, pe_pgrs-gene*s holds a key to understanding virulence of Mtb^41,42^ and in this analysis, these areas have been omitted due to difficulties with sequencing them by the method used. This limitation could be overcome in the future by using long-read sequencing, such as PacBio or Oxford Nanopore MinION.

In conclusion, our data show that the success of C2 cannot be readily attributed to acquisition of antibiotic resistance or demographic factors, such as nationalities and ethnicities. We suggest that bacterial genetic factors (such as polymorphisms in genes related to virulence), likely in combination with poor TB control, are the main contributors to the success of C2. Our identification of C2 specific polymorphisms in genes related to virulence constitute a valuable basis for studying Mtb virulence, and our results facilitate comparative studies as more sequencing data sets from outbreak strains becomes available.

## Materials and Methods

### Study population

Over the study period, 1992–2014, the incidence of TB in Denmark ranged from 6–12 (7.1 in 2014) per 100,000^43–45^. In this period, a total of 9,501 Danish TB cases were culture-positive, from which 94% had at least one Mtb isolate successfully genotyped, by RFLP until 2006 or by MIRU-VNTR after 2006. Out of all typed isolates, 61% clustered with other cases [2,415 DB; 396 GB; 2,653 FB], and 39% remained un-clustered [972 DB; 41 GB; 2481 FB]. Isolates from 989 cases [694 DB; 240 GB; 55 FB] were assigned to the C2 outbreak with either IS6110-RFLP or MIRU-VNTR typing, comprising 18% of all clustered Danish cases over the 23-year sampling period.

### Initial processing of strains

All culture positive strains at the International Reference Laboratory of Mycobacteriology (IRLM), a biosafety level 3 (BSL-3) certified laboratory, are subjected to testing of antimicrobial resistance and genotyping. Genotypic susceptibility testing was initially done with a Line Probe Assay from Hain (Nehren, Germany), testing for rifampicin (RMP) and isoniazid (INH). Phenotypic drug susceptibility testing was done by sub-culturing in Dubos medium with 0.045% tween 80 (SSI Diagnostika, Hilleroed, Denmark) and incubating at 37 °C. After 2 weeks of incubation, 100 µL of positive culture media was inoculated on a blood agar plate incubated at 35 °C and was checked for growth of other microorganism after 48 hours and microscopy was performed. If no contaminants were found, 500 µL were transferred to a MGIT tube, and after 2–3 days, when positive, the bacterial concentrations in liquid media were adjusted to equal densities at 580 nm by adding Dubos-Tween. One mL of positive broth was diluted in 4 mL of sterile saline and 0.5 mL was used for each drug containing tube. For the growth control (GC) tube in INH, RMP, and EMB test 0.5 mL of 1:100 dilution was used. For the GC tube in PZA test 0.5 mL of 1:10 dilution was used. All drugs were provided by the manufacturer and used in the concentrations 0.1 mg/L for INH, 1.0 mg/L for RMP, 5.0 mg/L for EMB and 100 mg/L for PZA.

Subtyping was done with RFLP as described elsewhere^46^ and MIRU-VNTR DNA extraction was performed directly from stock and MIRU-VNTR typing performed as described by Supply *et al*.^47^ with a commercial kit (Genoscreen, Lille, France) and processed with a 48-capillary ABI 3730 DNA Analyzer (Applied Biosystems, CA, USA). The MIRU-VNTR allele assignation was performed using GeneMapper software (Applied Biosystems, CA, USA) or BioNumerics (Applied Maths, Sint-Martens-Latem, Belgium).

### WGS

DNA isolations on the first 200 isolates were done as previously described^48^ and subsequently, in order to sequence the remaining almost 800 isolates more quickly, as described by Votintseva *et al*.^49^. In brief, 1 mL of a culture enriched in MGIT was centrifuged at 13,000 RPM for 10 min., the supernatant removed and pellet resuspended in 400 µL water, heat inactivated at 95 °C for 15 min and sonicated 15 min at 65 °C. The supernatant was mixed with 1/10^th^ volume 3 M sodium acetate and 2 volumes of ice-cold 96% EtOH, vortexed and incubated at −20 °C for 1 h. After centrifugation, the pellet was washed with 70% EtOH and subsequently air-dried and re-suspended in 50 µl Tris-EDTA (TE) buffer by heating. Supernatant was transferred to a plate and cleaned with AMPure XP beads according to protocol.

Library preparation and variant calling was performed as previously described^11^. High quality single nucleotide polymorphism (SNP) positions were retained if at least one sample had at least four reads coverage in each direction and a SNP frequency of at least 85%. To robustly identify universally conserved and abundant SNPs in C2, we randomly subsampled 500 out of the 952 strains multiple times (*n* = 10) and only retained positions identified as universally conserved (monomorphic) or abundant (present in >4 samples) more than once. The presence of universally conserved- and abundant SNPs was then verified for all samples using less stringent criteria (minimum read coverage of 3 and a minimum SNP frequency of 70%). The synonymous substitution rate per site to the non-synonymous substitution rate per site (dN/dS ratio) was calculated as previously described^26^.

All the genes in which we found monomorphic non-synonymous (NS) SNPs where used in a literature search, looking for reports of these genes being involved in virulence.

### Data availability

The data generated during the current study are available in the EMBL-EBI European Nucleotide Archive (ENA) under study accession PRJEB20214. https://www.ebi.ac.uk/ena/data/view/PRJEB20214

### Ethical considerations

This study was approved by the Danish Data Protection Agency (Jnr. 2012-54-0100). In accordance with Danish law, observational studies performed in Denmark do not need approval from the Medical Ethics Committee or written consent from subjects. All analyses are presented anonymously.

## Electronic supplementary material

Supplementary Table S1
